# “Tell Me What My Job Is”: A Qualitative Exploration of the Experiences of Autistic Academic Staff Working in Higher Education in Ireland

**DOI:** 10.1177/13623613261440799

**Published:** 2026-04-24

**Authors:** Neil Kenny, Alison Doyle, Claire O’Neill, Jessica K. Doyle, Jane O’Kelly, Fiona Earley, Stuart Neilson

**Affiliations:** 1 Institute of Education, Dublin City University, Ireland; 2 Caerus Education, Ireland; 3 University College Cork, Ireland; 4 Independent Researcher, Ireland

**Keywords:** academics, autism, higher education, neurodiversity, space, workplace inclusion

## Abstract

This co-produced study explores the experiences of autistic staff working in higher education in Ireland, a group largely overlooked in existing research. While much attention has been given to autistic students, little research has explored how autistic academics navigate their professional roles. This study investigates the challenges and strengths autistic staff encounter within academic environments. Eleven autistic participants took part in semi-structured interviews, conducted flexibly to respect individual preferences and communication needs. Data were analysed using the Reflexive Thematic Analysis. Four key themes emerged: (1) Discovering being autistic, (2) Role ambiguity and institutional invisibility, (3) Stress, burnout, and workplace unpredictability, and (4) Autistic strengths. Participants described strengths such as hyper-focus, problem-solving, and deep commitment to teaching while also highlighting barriers such as unclear expectations, a lack of visibility, and high emotional labour. The findings underscore the need for more inclusive institutional practices that reduce the pressure to mask and protect against burnout. Supporting autistic staff through affirming environments not only enables individuals to thrive but also enriches the wider educational community by fostering diverse approaches to teaching, learning, and communication.

**Lay Abstract:**

This study explores the experiences of autistic people who work as academic staff in universities. Autistic staff often face challenges such as unclear job expectations, misunderstandings from colleagues, and barriers to being open about their identity. Through interviews, we learned that many autistic academics care deeply about their work but feel unsupported in environments that reward constant social interaction, speed, and competition. Despite this, participants found creative ways to make space for themselves and others. This research helps us understand what autistic staff need to thrive in universities and shows why workplaces should value different ways of thinking, working, and communicating. Making these changes could benefit not only autistic staff but the wider academic community as well.

## Introduction

Despite growing interest in the experiences of autistic students in higher education (Gormley et al., 2023), the experiences of autistic academics remain largely overlooked, particularly in teaching and research roles (Jones, 2023a). This omission reflects systemic epistemic injustice (Fricker, 2007), where autistic voices are excluded from knowledge production and institutional dynamics shaping academic labour remain underexamined.

Historically, research exploring autistic neurotypes has been shaped by the medical model, framing autistic people as disordered or deficient (Walker & Raymaker, 2021). This has contributed to a narrow understanding of autistic experience that overlooks how high levels of ability can co-exist with complex support needs (Kenny & Doyle, 2024). As a result, the perspectives and experiences of autistic academics have been largely excluded from the literature. A recent scoping review identified only five studies focusing specifically on autistic academic staff between January 2007 and June 2021 (Gormley et al., 2023). Importantly, they also noted that no previous published studies explored the lived experience of this cohort in Ireland (Gormley et al., 2023). Existing research on autistic academics has been conducted primarily in the United Kingdom, the United States, and Australia, contexts characterised by more established disability workplace accommodation processes, and extensive institutional research on neurodiversity in higher education (e.g., Brown & Leigh, 2018; Jones, 2023b; Wood & Happé, 2021).

This study responds to that gap, drawing on the Autistic SPACE framework (Doherty et al., 2023) to explore how environmental and social conditions shape autistic academics’ working lives. (SPACE in this framework is an acronym standing for Sensory needs, Predictability, Acceptance, Communication and Empathy) Grounded in a rights-based perspective, it affirms that equitable academic employment is a fundamental right.

### Autistic Neurology

Autistic neurology is characterised by distinctive perceptual mechanisms, sensory processing and self-regulating, monotropic cognitive styles, distinct communication patterns, and different ways of connecting (Hartman et al., 2023; Walker, 2021). The neurodiversity paradigm recognises these as valuable differences rather than deficits (Fletcher-Watson & Happé, 2019; Walker, 2021). However, while the majority of autistic individuals do not have a co-occurring intellectual disability (Shaw, 2025), their rates of employment participation remain low, with only 22% in full-time work in the United Kingdom (Sparkes et al., 2022). Ireland’s disability employment gap is double the Organisation for Economic Co-operation and Development (OECD) average (McCoy et al., 2025; Scanlon & Doyle, 2021; Thewissen et al., 2021), and no statistics exist for autistic engagement in employment in Ireland (O’Kelly et al., 2024). These figures suggest systemic and societal barriers restricting opportunities (McCoy et al., 2025).

Barriers to autistic flourishing (Pellicano & Heyworth, 2023) include masking, burnout, and minority stress (Botha & Frost, 2020; Pearson & Rose, 2021). Masking—suppressing autistic traits to fit neurotypical expectations (Pearson & Rose, 2021)—may help navigate social contexts but is linked to stress, exhaustion, and mental health difficulties. Milton’s (2012) Double Empathy problem reframes social difficulties as reciprocal miscommunication, emphasising that employment inclusion must be rooted in dignity, rights, and social justice (O’Kelly et al., 2024).

### Autistic Employment in Higher Education

There is still limited understanding of autistic individuals working in academic roles in higher education, such as people pursuing postgraduate study, working, teaching, or in research roles (Gormley et al., 2023; Jones, 2023a). Limited research in this area may reflect a reluctance to disclose an autism diagnosis due to fears of stigma and negative repercussions (Thompson-Hodgetts et al., 2020). Disclosure can be a double-edged sword (Jones, 2023b; Thompson-Hodgetts et al., 2020). Botha and Frost (2020) and Jones (2023b) advocate for institutional policies ensuring confidentiality and fostering understanding and a supportive workplace culture.

Brown and Leigh (2018) highlight how a lack of appropriate accommodations within rigid academic systems can lead to sensory overload, and social demands contribute to isolation due to differing communication or social expectations (O’Neill & Kenny, 2023; Pearson & Rose, 2021). Botha and Frost (2020) link this to minority stress—the chronic social stress felt by a minority group othered by the dominant group (Helsen et al., 2022; Meyer, 1995). This stress can exacerbate burnout, especially when coupled with the high demands and limited support commonly found in academia (O’Neill & Kenny, 2023).

Autistic burnout is a serious concern. Mental, emotional, and physical exhaustion, loss of skills, and increased sensory sensitivity (Higgins et al., 2021; Phung et al., 2021) stem from sustained masking and institutional pressures (Higgins et al., 2021). The Burnout, Isolation, and Mental Strain (BIMS) model explains how institutional factors drive burnout in autistic academics (Botha & Frost, 2020; O’Neill & Kenny, 2023; Phung et al., 2021). Jones (2023b) identifies effective targeted interventions, including flexible work arrangements, autistic-specific accommodations, and mentorship programmes.

The Irish higher education context differs in several important respects. While Ireland has made significant advances in disability inclusion for students, institutional policies and supports for disabled and autistic staff remain comparatively underdeveloped, and national data on autistic employment are notably absent (O’Kelly et al., 2024). Disclosure is often negotiated within relatively small academic communities, where concerns about visibility, precarity, and reputational risk may be heightened (Jones, 2023a; O’Neill & Kenny, 2023). Irish higher education has also undergone significant reorganisation and restructuring in recent years, further adding to institutional instability (O’Kelly et al., 2024).

### Autistic SPACE Framework

While existing literature has identified barriers such as masking, burnout, and minority stress among autistic people in employment, these issues are often examined in isolation or framed primarily at the level of individual adjustment. There remains a need for an integrative framework that foregrounds the interaction between autistic people and their working environments (Neilson et al., 2025; Wong, 2023) and that can support both analytic understanding and systemic change. In this study, we draw on the Autistic SPACE framework (Doherty et al., 2023), as it explicitly centres environmental, relational, and organisational conditions shaping autistic experience, rather than locating difficulty within the individual.

**Figure 1.**
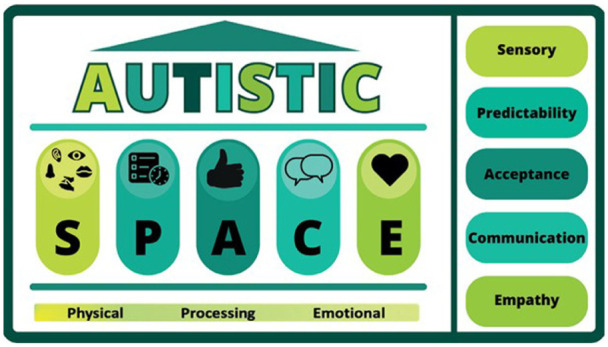
Autistic SPACE framework (Doherty et al., 2023).

Autistic SPACE was originally conceived in 1992 (Sinclair, 1993) and subsequently developed by Doherty and her colleagues (2023) into a framework bridging theory and practice when prioritising autistic needs. The framework (Figure 1) is centred around an acronym capturing the core needs of heterogeneous autistic individuals, thus facilitating a more effective and equitable engagement with systems and services. SPACE is a practical and consistent tool that incorporates an understanding of and a facilitation of autistic Sensory needs, Predictability, Acceptance, Communication and Empathy. Although developed in the context of healthcare settings, the framework is flexible and efficacious in other systems, with notable examples being education (McGoldrick et al., 2025) and adult settings (Prendeville, 2025).

The Autistic SPACE framework considers the physical, processing, and emotional environment in cultivating work environments in higher education where autistic staff experience belonging and acceptance. The Autistic SPACE framework (Doherty et al., 2023) is particularly well suited to higher education contexts, where sensory environments, role ambiguity, communication norms, and institutional cultures play a central role in shaping academic work and well-being (Jones, 2023b). It, therefore, offers a coherent lens through which to examine autistic academics’ experiences while also informing institutional practices and policy.

### Current Study

This study examines the lived experiences of autistic academic staff working in higher education in Ireland, thereby addressing the identified gap in Irish and international literature. The Autistic SPACE framework (Doherty et al., 2023) informed the overall design of the study by orienting attention towards environmental, relational, and institutional factors shaping autistic academics’ working lives. This aligned with the study’s rights-based and neurodiversity-affirming approach, supporting a focus on how academic systems enable or constrain autistic flourishing. Beyond supporting analytic understanding, Autistic SPACE offers a practical framework (McGoldrick et al., 2025) through which higher education institutions (HEIs) can reflect on and redesign environments, policies, and practices to better support autistic staff.

The study specifically addresses the following research questions:

**Research Question 1 (RQ1):** What are the experiences of autistic academic staff working in Irish higher education?
**Research Question 2 (RQ2):** What strengths and challenges do autistic staff encounter in these settings?

## Methodology

This study adopted a co-produced, participatory research approach, grounded in collaborative practices between autistic and non-autistic researchers. Co-production was embedded throughout, from initial design and development of research instruments to data collection, analysis, and dissemination, ensuring shared decision-making and inclusion of autistic perspectives (Nicolaidis et al., 2019).

The study foregrounded power-sharing, inclusivity, and mutual respect. Using the concept of “choice points” (Kenny et al., 2023), the team made joint decisions at key moments (Fletcher-Watson et al., 2019; Kenny et al., 2023; Stark et al., 2021). In practice, this meant differing levels of participation and activity at stages of the research process, with agreement that some members may take leading roles in aspects of research, analysis, or writing stages.

### Epistemological Stance

A critical realist epistemology underpins the current study, which recognises that autistic academics’ experiences are shaped by real social and structural conditions while understanding that knowledge of those experiences is interpretively constructed (Kourti, 2021). This stance aligns with the neurodiversity paradigm, which views autism as a natural variation rather than a deficit (Fletcher-Watson et al., 2019). Together they support an analysis that values autistic expertise, attends to structural barriers, and remains reflexive about how researcher perspectives shape interpretation (Kourti, 2021).

#### Community Involvement, Co-Production, and Researcher Positionality

This study was co-produced by a mixed team of autistic and non-autistic researchers, with co-production embedded across all stages of the research process. Aligned with the neurodiversity paradigm (Walker, 2021) and a critical realist framework (Kourti, 2021), we acknowledge that our lived experiences of autism and academic work informed the questions we asked, the emphasis on systemic barriers, and the reflexive thematic analysis. This shaped an autism-positive design, with careful attention to language, accessibility, and communication modes.

Autistic researchers were involved in study design, development of research materials, data collection, analysis, and interpretation, with shared decision-making at key points (see Table 1 for specific information). Co-production was operationalised through flexible participation, role negotiation, and collective reflexive discussion, rather than assuming uniform involvement at all stages (Kenny et al., 2024; Stark et al., 2021).

**Table 1.** Co-Production Across Stages of the Research Process.

Research stage	Co-production activities
Study design	Joint development of research aims, discussion of ethical considerations, and selection of participatory methods
Materials development	Collaborative development and review of participant information, consent materials, and interview topic guide
Data collection	Interviews conducted by autistic and non-autistic researchers; flexible formats negotiated with participants
Data analysis	Initial coding led by one researcher; themes reviewed, refined, and interpreted collaboratively
Interpretation and dissemination	Reflexive discussion of findings; agreement on framing, language, and implications

### Methods

#### Participant Recruitment

Staff in Irish HEIs were recruited through convenience sampling. Invitations were sent in writing to Registrars and School Heads and circulated via academic and autistic networks, social media, and a project website with study details and researcher bios. Ethical and General Data Protection Regulation (GDPR)-compliant data handling was outlined. Inclusion required employment in an Irish HEI with at least one semester of teaching experience, self-identification or formal autism diagnosis, and willingness to participate.

Eighteen people responded, 14 met the criteria and consented, and 11 completed the interviews. Participants who withdrew after consenting are included in Table 2 for transparency in reporting recruitment and participation pathways; in line with ethical requirements, all data from these participants were excluded from analysis. Recruitment coincided with examination periods, which may have limited responses.

**Table 2.** Participant Characteristics.

Participant ID	Gender/sex	Role category	Participation
P1	Female	Academic Staff	Withdrew
P2	Female	Academic Staff	Zoom Interview
P3	Female	Academic Staff	Zoom Interview
P4	Female	Research Staff	Zoom Interview
P5	Female	Academic Staff and Industry	Zoom Interview
P6	Female	Academic Staff	Zoom Interview
P7	Male	Academic Staff	Zoom Interview
P8	Female	Research Staff	Withdrew
P9	Male	Academic Staff	Withdrew
P10	Female	Academic Staff	Zoom Interview
P11	Male	Academic Staff	Zoom Interview
P12	Female	Academic Staff	Zoom Interview
P13	Male	Research Staff	Zoom Interview
P14	Female	Research Staff	Zoom Interview

*Note.* This table displays participant gender/sex, role category, and participation format.

#### Procedures

All team members contributed to the interviewing and analysis. Each participant worked with one allocated researcher in a three-step flexible process tailored to individual communication preferences:

1. introductory meeting to explain the project and agree on interview format (single or multiple sessions, online or text-based);
2. interview as agreed;
3. participant review of the transcript and emerging codes, with feedback incorporated into data analysis.

While interviews remained semi-structured and participant-led, the Autistic SPACE framework acted as a sensitising guide during the development of the topic guide. Questions were designed to invite reflection on sensory environments, clarity of roles and expectations, communication practices, experiences of acceptance, and relational dynamics within academic settings, without explicitly naming the framework to participants.

Some overlap across topic areas (particularly in relation to support, challenges, and disclosure) was intentional, as these experiences cut across different aspects of academic work and were explored flexibly during interviews. Questions were therefore organised by domain rather than treated as mutually exclusive, consistent with a flexible, participant-led qualitative approach.

Interviews were conducted by autistic and non-autistic researchers, guided by five themes: academic work, teaching support, barriers, strengths, and recommendations (see Appendix 1). Data were securely uploaded to an encrypted university platform in line with data protection policy and anonymised; pseudonyms were assigned.

### Data Analysis and Credibility

Data were analysed using reflexive thematic analysis (Braun & Clarke, 2021) through a collaborative process, with themes generated from participants’ accounts rather than predefined categories. The Autistic SPACE framework was subsequently used as an interpretive lens to support sense-making and to examine how the identified themes related to environmental and relational conditions within higher education.

One researcher led initial coding, with collaborative review and refinement of themes by the full research team. The team reviewed themes collectively and applied the final framework to confirm coherence. This approach ensured analytic flexibility while enabling the findings to be situated within a framework with clear relevance for institutional practice.

Credibility was supported by shared memos in a shared institutional Google Drive folder, allowing all researchers to read, comment on, and build upon one another’s reflection in addition to peer debriefing during Zoom-hosted research meetings. An audit trail documented coding decisions, theme development, and iterative returns to the data to ensure interpretations remained grounded and reflexive.

Final themes were reviewed by the full research teams. Table 3 shows an outline of the processes followed at each stage of the data analysis process.

**Table 3.** Reflective Thematic Analysis Processes Followed.

Step number	Stage of TA	Description of action
1	Familiarisation with the data	Researchers immerse themselves in the dataset by reading and re-reading transcripts, listening to audio if available, and noting early impressions or analytic memos.
2	Generating initial codes	Working inductively, the lead analyst coded each transcript line-by-line, capturing both explicit statements (e.g., “role ambiguity”) and underlying meanings such as institutional ableism or masking. Codes were documented in an organised coding framework with illustrative quotations.
3	Constructing initial themes	Codes are organised into broader patterns of meaning, potential themes, by clustering related codes and exploring conceptual links. These early themes were shared with the wider team for comment.
4	Reviewing and developing themes	All team members, autistic and non-autistic, met to review the candidate themes against the full dataset, debating overlaps, refining boundaries, and merging or splitting themes where needed.
5	Defining and naming themes	Through iterative team discussions and memo-writing, each theme’s core narrative was clarified and named to convey the central organising concept (e.g., “Role Ambiguity and Institutional Invisibility”).
6	Writing the report	The final analysis was written collaboratively, weaving vivid participant quotations with interpretation and situating findings within the Autistic SPACE framework and neurodiversity paradigm.

### Ethics

Ethical approval was granted by the University Research Ethics Committee (Reference No. DCUREC/2021/166). All participants provided written informed consent. All participants provided written informed consent. Data were handled in accordance with the GDPR, the European Union framework governing the lawful, secure, and confidential processing of personal data.

### Findings

**Figure 2.**
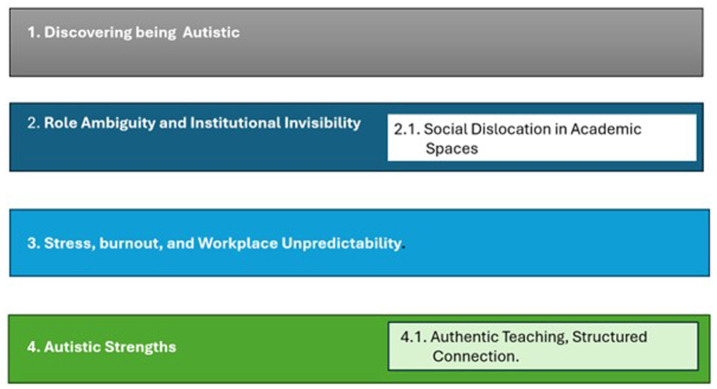
Themes and subthemes.

This section will present the findings relating to the lived experiences of autistic individuals working in higher education in Ireland. Four themes and two associated subthemes emerged from the analysis, which are represented in Figure 2.

#### Discovering Being Autistic

For participants in this study, becoming aware of their autistic status had a transformative impact on their lives, allowing them to understand and validate their experiences. Participant 13 reflected:

Getting a diagnosis definitely clarified for me why I do certain things and why I avoid teaching certain topics, and even the way I communicate with students. The chaos made a lot more sense.

Other participants echoed the importance of access to an autism diagnosis in supporting self-understanding. Participant 13 described a “lightbulb moment” during an autism training session for staff, which prompted reflection on their experiences at work. This recognition often led to a reassessment of both personal and professional relationships and developing awareness of social mismatches or negative experiences. Participant 11 noted,

Since I was diagnosed, I’ve effectively ditched most of the people I grew up with . . . because I realised that my difficulties in dealing with them were part of my autistic profile.

Diagnosis also relieved internalised stigma. One participant reflected, “When I got my diagnosis, I was elated because it made so much sense of everything” (P6). Another described the transformative nature of their diagnosis: “It took away all the horrible labels I had for myself” (P3).

I couldn’t tell anybody because I just thought I was weird. I’ve said to myself many times, “Why are you so weird?” Now I have a name for it, and it’s kind of good. I’m very verbal, and people often question my autism because of how I present myself. They say, “What do you mean you’re autistic? Look at what you do, look at how you do it, you can’t be.” (P5)

Following their diagnosis, Participant 11 described allowing themselves to unmask and embrace their authentic selves: “I’ve become quite blunt and frank, an autistic person being blunt, there’s a thing!”

The diagnosis also helped participants reconcile how others perceived them. Participant 14 recounted, “In my previous workplace . . . they’d say ‘oh it’s great, like, you’re so good at masking,’ and I said ‘excuse me, this is who I am.’”

#### Role Ambiguity and Institutional Invisibility

The participating autistic academics reported that their roles often lack clear boundaries and structure, with Participant 10 stating, “The diffuseness, I suppose, the diffuseness of the role, is something that I find really challenging. . . constantly bombarded by emails and demands, while I’m trying to research.” Balancing multiple demands can become overwhelming, especially as “other things go on the back-burner” until they demand immediate focus. This diffuseness can be challenging, particularly for autistic individuals who benefit from predictability and well-defined responsibilities.

Vague job responsibilities make it difficult to establish boundaries or request support for something as abstract as role clarity. This can also complicate daily organisation, with Participant 13 describing the challenge of keeping track of commitments, explaining:

[it’s] “the organisational stuff, it’s not even the teaching. . .The most challenging thing, of being an academic with autism, . . . is trying to be organised to be in the right place at the right time, plenty of times, I get texts from students that I’m supposed to be somewhere else . . . .”

Participant 4 also echoed this point, describing themselves as “time blind” but also as a “bit of a perfectionist” which added difficulty to the role because “it would probably take me three times as long as anybody anyway . . . diligent but erratic.”

A further challenge concerned the difficulty in requesting accommodations, especially for role clarification. Participant 6 explained, “The accommodations that I could do with are not straightforward, . . . How do you ask for ‘tell me what my job is’?” Participant 5 explained, “just trying to behave in a neurotypical world, to be in a neurotypical world” (P5). Participant 6 described the dissonance of encountering professionally the same barriers they researched academically:

So, it’s funny to experience it in real life, you know, when this is the kind of thing you research about, and the kind of barriers that people face and then it’s here, confronting me. In my own workplace. (P6)

##### Social Dislocation in Academic Spaces

Participating autistic academics often encountered misunderstanding and exclusion in their professional relationships. Reflecting on previous jobs, Participant 4 recalls being described as “odd” or “really weird” by colleagues, highlighting how neurotypical coworkers may misinterpret their natural differences. Such bidirectional misunderstandings can lead to uncomfortable or even hostile interactions. Participant 4 recounts an incident where a colleague said “. . . my brain stops working when you talk.” The incident was later framed as a form of “bullying.” Participant 11 describes the reluctance to disclose their autistic status, as the emotional weight of disclosure can be significant.

I find it hard to understand how you could go and say “I’m experiencing something that is essentially bullying, but it’s happening because the person doesn’t know I’m autistic and are doing something, that would be quite normal for neurotypicals to do to each other” . . . and of course then you would have to disclose. It would take a huge amount to disclose to my colleagues that I’m autistic. (P11)

The efforts to hide or monitor themselves in the presence of others can have negative impacts, with Participant 14 describing the effort and negative self-judgement of this process: “I’m already impostering and a fraud, a fraud in my own mind,” reflecting their negative view of themselves as inauthentic or “a fraud” due to ongoing self-monitoring or masking over a long period of time.

For many autistic academics, their commitment to fairness and authenticity is central to their work style, but it may not align with the typical values tied to career progression. Participant 10 reflected on prioritising being “kind and fair and transparent,” noting that this could be a disadvantage in an environment that often rewards assertiveness and self-promotion. Participant 4 observes that avoiding “posturing,” could result in contributions being overlooked.

This lack of recognition can have an emotional impact. Participant 6 reflected on encountering the same barriers they studied academically within their own workplace. Criticism was described as particularly difficult, with Participant 14 explaining that they: “take the feedback to heart,” leading them to question their suitability for academia. Participant 6 described withdrawing socially when masking became difficult to sustain:

I did try [undergraduate career] and this happens a lot in my life, I’ll start really strongly but then it’s like with the masking, I’ll start fine and the, it’s hard to keep it up, it’s hard to maintain the mask, and if you’re stressed out, the mask will slip . . . and then, you know, I’d either just avoid social situation, if I didn’t feel able for it or I’d go and just maybe make, well, I feel, a bit of a fool out of myself, drink too much as well, because I was so anxious. (P6)

#### Stress, Burnout, and Workplace Unpredictability

Participants noted the expectation and culture in some areas of academia to meet high-demand workloads and high-performance expectations. Workplace stress was a recurring issue for participants, often linked to sensory and social demands within academic environments. Participant 4 described the exhausting effort of simply staying engaged at work: “Access fatigue” means “having to exert additional effort just to engage on the same level as peers”:

I can’t work for the intense period of time that some of my colleagues can, and being online has been difficult for that . . . people were working at 10, 11, 12 at night on it, and it was kind of an expectation we do that, and I find that I can’t do that solidly, I need to step away. (P12)

Some participants discussed how these workload issues or pressures interacted with their autistic neurology in ways that may differ from their non-autistic colleagues. Indeed, some of these accounts showed significant levels of self-understanding and awareness of how they negotiate these workload-related stresses:

Autistic inertia, if I have a class at 3 p.m. I’ll sit waiting for the class, nothing else gets done, or I could be preparing for the class, and thinking about the class . . . I may not sleep the night before because I’ll be thinking about the class. I think everybody’s challenged by it, but I think I’m challenged by it in different ways. I can shift focus to something but it’s shifting the focus away from something, that’s more challenging for me so . . . I do a task it’s hard then to get back from it. (P10)

One participant described compensating for their inability to cope by relying heavily on routines and lists, craving predictability and “sameness” which is “whats good about university” (P5):

I was compensating for that lack of being able to cope . . . having lists for everything, needing that routine, like craving that routine, and predictability, and order on things. (P3)

However, the pressure to meet student expectations while staying true to oneself created additional strain: “I put in a 12 hour day for a 20 minute worth of a demonstration” (P3). This emphasises how deeply some participants prepare themselves for teaching in order to support a feeling of predictability, supporting confidence and mastery of the subject of their teaching:

Everything I teach, I fully understand . . . I’m completely absorbed in it . . . I’ve never, ever been caught out. (P11)

Participant 14 noted that their challenges with managing work-related stress are not recognised by others due to their perceived abilities to work in higher education settings:

the GP would have been very dismissive, [saying] but sure, you’re working, what more do you want? Like what do you want that label for? . . . And I’m like, well the fact that I’m in here every 6 months burnt out with anxiety. (P14)

Participants linked this to experiencing of autistic burnout from the prolonged effort of camouflaging or masking autistic traits to fit into neurotypical environments. Participant 14 described themselves as “. . . a fraud in my own mind” due to self-monitoring themselves over a long period of time, something they felt would eventually lead to a “payoff” of burnout or anxiety (P14). Another highlighted the exhaustion from managing social interactions and the subsequent depression (P6).

#### Autistic Strengths

Autistic academics, particularly autistic individuals, frequently identify hyper-focus as one of their most valuable strengths, often leading to exceptional productivity and innovative outcomes. Participant 3 described their ability to “ask those unusual questions . . . seeing things differently.” This participant also identified their exacting and diligent approach as a positive aspect they brought to their work, particularly in research settings:

I’ll show up and do the work . . . D’you know that kind of way? And I won’t do it half arsed . . . So that kind of thoroughness, may be, kind of . . . professionalism, I think the ability to kind of see maybe kind of like unusual connections between things or asking those unusual questions of something. is definitely a strength just from seeing things differently. (P3)

Participant 2 observed that “some parts of the job lend themselves well to . . . hyper-focus . . . this kind of like, drilling down into, I love that, very hyper-focused.” Similarly, Participant 12 noted their strength in identifying patterns and anticipating challenges, explaining how they “dig down” into tasks in ways others might not. Participant 10 described their “monotropic brain,” which enabled them to “engage intensively in focus on one particular thing.”

So, the research thing is a real clearer thing of that, because we like to go deep, deep, deep into something, you know, and think about it all the time, and then come up with a new finding or whatever. And that’s what autistic people do, and that should be celebrated in universities. If you go back and look at, see all the Nobel Prize winners, you know, I’d say 70% of them, are probably autistic! (P5)

The academic environment also provides a space where autistic individuals can pursue their passions, often turning their special interests into academic careers. Participant 3 highlighted the role of predictability in enhancing focus, stating, “if you get certain predictability, it can be very, very supportive.” Participant 5 described pursuing a PhD as “autism-friendly” as it allows for in-depth work in an area of special interest, often pursued in solitude. Participant 4 emphasised the joy of connecting with others who share their passion, stating, “I love the fact that I get to talk to other people who are really passionate about it and have a really high level of knowledge.”

Problem-solving is another area where autistic academics excel, often bringing unique insights to their work. Their meticulous attention to detail further enhances their contributions. Participant 10 reflected on this, stating, “pattern-spotting, spending hours cataloguing information and data is certainly helpful in my research.”

Participants could identify the value their neurology and ways of working brought to their institutions. Participant 11 shared how their analytical approach helped them manage complex systems that other colleagues found very challenging, noting, “I first familiarised myself with the software packages involved, jumped straight in.”

they talk about your autistic special interest? Like mine is managing . . . figuring out how to get shit done, that’s my special skill, how can I navigate stuff that I initially think I can’t do, how can I navigate and do it? (P14).

Participant 12 highlighted their “very, very forensic” approach as particularly beneficial in teaching and administration. Participant 3 framed their meticulousness as an ethical commitment, describing it as “that kind of thoroughness . . . professionalism . . . there’s like almost an ethical, like a vocation.”

##### Authentic Teaching, Structured Connection

Teaching and engaging with students emerged as an area of pride and reward for many of the participants. For example, Participant 10 stated: “I love my job, there’s nothing else I would rather do, . . . I find teaching and classroom work, working with the students so hugely rewarding” (P10). However, a nuanced and detailed account of their experience of the teaching aspects emerged, which reflected the balancing act many autistic academics perform, navigating their preferred communication styles while meeting the expectations of higher education environments.

Their teaching approaches often reflected clarity, structure, and dedication: One participant emphasised, “My approach is usually very clear and logical, and it’s very easy to follow, so I will do things in a fairly structured way” (P3). Despite these strengths, the social aspects of teaching were more difficult for some participants. Participant 3 noted,

I could lecture all day. . . but it’s when you get to the kind of seminars, the more interactive side with students. . . I’m a little bit, not like standoffish, I’m chatty, but I do give them a lot of clarity so that they’re not coming near me.

A preference for structure and predictability is a recurring theme in the experiences of autistic staff. Over-preparation is a common strategy, as one participant described: “I think the masking side of things . . . I think because I over-prepare so much it doesn’t really happen” (P3). This “over-preparation” serves as a coping mechanism to manage the unpredictability of the classroom environment.

The relationship between autistic staff and their students was often marked by deep care and empathy, “I very much see it as that, you know, nurturing ideas, nurturing critical thinking, nurturing the students and their own sense and self-confidence” (P10). This nurturing approach, while deeply fulfilling for some, can also lead to challenges when students do not meet expectations, with Participant 10 suggesting “. . . hyper-empathy can be a bit of a challenge” and referencing others accusing her of “mothering the students.” Other participants felt a connection to students who were vulnerable or different, with Participant 12 remarking, “I have always had a sense of . . . the underdog, and supporting someone who is, has a difference.” Participants felt this contrasted with the approach they observe among some colleagues who they suggest “take very austere, formal, kind of attitudes towards the students” and just try to “intimidate the students . . . which I think is morally wrong” (P11).

## Discussion

This study highlights the nuanced experiences of autistic academic staff in Irish higher education, addressing the complexities of their roles, experiences, strengths, and challenges. Two questions guided the study: (1) What are the experiences of autistic academic staff working in Irish higher education? and (2) What strengths and challenges do autistic staff encounter in these settings? This discussion interprets the findings through the Autistic SPACE framework (Doherty et al., 2023), using it as a lens to examine how environmental, relational, and institutional conditions shape autistic academics’ experiences of work. Rather than mapping themes deductively onto predefined categories, SPACE is used here to support synthesis across themes and to consider implications for higher education policy and practice.

### Discovering Being Autistic

Diagnosis emerges as a transformative milestone consistent with previous research (Lewis, 2017). Recognising their autistic identity enabled participant self-understanding (Huang et al., 2022), reduced internalised stigma (Botha & Frost, 2020), and reframed past experiences (Leedham et al., 2020; Wood & Happé, 2021). In this sense, diagnosis functioned not merely as a clinical label but as a form of epistemic justice (Fricker, 2007), legitimising participants’ lived experiences and countering deficit-based interpretations of their working lives.

Interpreted through the Autistic SPACE framework, these accounts highlight the centrality of Acceptance and Empathy in shaping autistic academics’ post-diagnostic experiences (Doherty et al., 2023). Participants’ descriptions of relief and increased self-compassion following diagnosis suggest that recognition and access to diagnosis align with previous research on relief and “unmasking” and improving well-being (Hull et al., 2021).

Importantly, this study extends existing literature by demonstrating that the benefits of diagnosis for autistic academics are not intrinsic to diagnosis itself but are mediated by institutional and cultural contexts. In contrast, delayed diagnosis prolongs exposure to environments lacking acceptance and prolongs the emotional burden of masking for participants’ post-diagnosis (Botha & Frost, 2020; Pearson & Rose, 2021). These reflections respond directly to the first research question.

### Role Ambiguity and Institutional Invisibility

Participants described role ambiguity as a persistent barrier to inclusion rather than a minor organisational difficulty. Consistent with previous research on autistic employment in higher education (Jones, 2023b), unclear expectations, diffuse responsibilities, and competing demands generated significant stress (O’Neill & Kenny, 2023). Participants’ accounts suggest that this ambiguity also functioned as a form of institutional invisibility, making their needs difficult to articulate, legitimise, or accommodate within academic structures.

From an Autistic SPACE perspective, difficulties related to Predictability and Communication were particularly salient (Doherty et al., 2023). Roles characterised by implicit expectations and informal norms conflicted with participants’ need for clarity around responsibilities and priorities, while the absence of explicit communication constrained their ability to identify or request appropriate support. As one participant noted, the challenge was not simply needing accommodation but needing someone to “tell me what my job is.”

These experiences point to institutional processes that assume neurotypical capacities for navigating ambiguity, contributing to structural ableism and epistemic exclusion (Fricker, 2007; Janse van Rensburg & Liang, 2023). In this context, role ambiguity operated as a stressor independent of overt discrimination, aligning with and extending minority stress perspectives (Botha & Frost, 2020). Addressing these issues requires structural approaches that improve role clarity and communication, rather than placing responsibility on individual disclosure or self-advocacy. Thus, directly addressing the second research question concerning systemic challenges.

### Social Disconnection in Academic Spaces

Participants described experiences of social disconnection and misunderstanding within academic workplaces, often linked to differences in communication style and expectations. These accounts align with Milton’s (2012) Double Empathy Problem, reframing social difficulties as bidirectional misattunements rather than individual deficits. Participants’ reflections suggest that behaviours perceived by colleagues as “odd” or inappropriate were frequently misinterpreted, contributing to feelings of exclusion and isolation.

From an Autistic SPACE perspective, Communication and Empathy were central to shaping autistic academics’ sense of belonging (Doherty et al., 2023). Participants described workplace interactions that generated uncertainty and stress, such as those characterised by indirect communication, implicit norms, and emotionally charged feedback. In some cases, misunderstandings escalated into experiences described as bullying (O’Neill & Kenny, 2023). In others, the anticipated emotional cost of disclosure deterred participants from seeking resolution (Romualdez et al., 2021). These accounts illustrate how limited empathic understanding and a lack of explicit communication can compound social disconnection, even in the absence of overt hostility.

Participants’ experiences further suggest that social disconnection in academia is shaped by institutional cultures that privilege neurotypical interactional norms, including informal networking and strategic self-promotion (Higgins et al., 2021; Jones, 2023b). Within such contexts, limitations in empathy and acceptance reinforce social precarity while also constraining opportunities for recognition and inclusion for autistic academics (Jones, 2023b; Romualdez et al., 2021b). Participants’ accounts point to the need for changes in how academic cultures support communication, relationships, and inclusion, rather than continued reliance on individual adaptation or disclosure.

### Stress, Burnout, and Workplace Unpredictability

Participants described persistent stress and exhaustion arising from academic environments marked by high demands, unpredictability, and limited opportunities for recovery. These accounts align with existing descriptions of autistic burnout as cumulative depletion linked to sustained masking and environmental strain (Higgins et al., 2021; Phung et al., 2021), with stress embedded in everyday academic practices rather than confined to periods of peak workload.

From an Autistic SPACE perspective, difficulties related to Predictability and Sensory load were particularly salient (Doherty et al., 2023). Shifting expectations, unclear timelines, and informal norms of constant availability generated ongoing anticipatory stress. Participants reported using strategies such as over-preparation and development of rigid routines as protective self-regulation responses, although these contributed to longer-term exhaustion (Higgins et al., 2021). These experiences reflect broader patterns of autistic burnout and align with the BIMS framework, which links emotional labour and environmental unpredictability to sustained burnout (Phung et al., 2021).

### Autistic Strengths

Participants noted traits like hyper-focus, pattern recognition, and ethical dedication to teaching, strengths previously acknowledged in the literature (Grove et al., 2016; Jones, 2023a). These accounts align with literature that emphasises the context-dependent nature of autistic strengths (Cherewick & Matergia, 2024; Pellicano & Heyworth, 2023). Participants noted that such strengths were most visible when they could work authentically, without sustained pressure to mask or adapt to implicit academic norms.

From an Autistic SPACE perspective, Acceptance and Predictability shaped whether these strengths could be enacted in practice (Doherty et al., 2023). Environments characterised by clarity, transparency, and relational trust enabled participants to teach and research in ways aligned with their values. Teaching philosophies marked by clarity, empathy, and fairness reflect neurodivergent pedagogical values (Baird, 2020; O’Neill & Kenny, 2023), with participants emphasising relational attunement, care, and ethical student engagement (O’Neill & Kenny, 2023; Wood & Happé, 2021).

Cherewick and Matergia (2024) conceptualise autistic strengths as context-dependent and relational, arguing that their positive impact on well-being emerges when environments and supports are intentionally designed to recognise and leverage them. These findings respond to both research questions by highlighting strengths while also signalling the need for deeper structural reform.

### Limitations and Future Recommendations

This study offers an in-depth qualitative exploration of autistic academics’ experiences within the Irish higher education context. The findings are not intended to be statistically generalisable but to provide transferable insights into how institutional environments shape inclusion, belonging, and agency.

While the focus on Ireland reflects a deliberate contribution to an under-researched context, institutional policies and cultures vary across higher education systems. Future research could examine how the experiences identified here compare across national and institutional contexts and further explore the application of the Autistic SPACE framework (Doherty et al., 2023) in supporting autistic academic staff.

The sample reflects those who were willing and able to participate in qualitative research and may not capture the experiences of autistic academics facing greater precarity. Further work is needed to examine how Autistic SPACE-informed approaches can be implemented and evaluated within HEIs.

## Conclusion

This co-produced study explored the personal views and experiences of autistic academic staff in Irish HEIs. While participants described challenges and barriers, their accounts revealed the substantial strengths autistic academics bring to higher education. Their hyper-focus, innovative problem-solving, and structured approaches to teaching exemplify their valuable contributions. However, institutional recognition of these attributes remains limited. By valuing these unique strengths, HEIs could enhance both the personal and professional experiences of autistic staff, fostering more inclusive academic communities. Drawing on Doherty et al.’s (2023) concept of Autistic SPACE, this research highlights the need for sensory-considerate, predictable, accepting, communicative, and empathetic environments.

### Implications of the Study

This study contributes to emerging scholarship on autistic academic staff by extending the Autistic SPACE (Doherty et al., 2023) framework beyond healthcare and student contexts, demonstrating its analytic value for understanding academic work and institutional culture. Through this lens, workplace experiences commonly attributed to individual disposition or preferences (such as uncertainty, exhaustion, and social disconnection, for example) are instead described in findings as shaped by environmental and organisational conditions. In particular, the analysis foregrounds how role ambiguity, implicit communication norms, and limited institutional acceptance function as structural barriers within academic roles. By centring autistic perspectives in all stages of the research and situating these experiences within a rights-based, neurodiversity-affirming framework, this study advances understanding of how HEIs can enable, rather than constrain, autistic flourishing at work.

## Appendix 1

### Interview Framework

1. Welcome
2. Overview of the Topic
3. Ground Rules
4. Opening Question

**Table:** Guiding Topic Framework and corresponding questions

Topic area	Guiding questions
Topic 1: Experiences of working in higher education (HE) setting	- How would you describe your experiences of working in HE as an autistic person? *Why do you say this? Can you give me an example?* - Was the experience of starting to work what you expected? *Can you give me an example?* Was there anything you would change or add? *Why?* - How were you supported in preparing to commence working in HE? - Have you disclosed that you are autistic and/or have a diagnosis of autism in your work setting? *Why is that? Are you happy with this situation?*
Topic 2: Experiences of supporting teaching and learning in higher education	- What are your experiences of supporting teaching and learning for your students? *Why do you say this? Can you give me an example?* - What do you like about this work? - What is a challenge? - What support is in place for this work? - What impact does being autistic have on your engagement with this work? - Do your students know that you are autistic? Why did you choose to tell them or not tell them? - What resources influence your work? - What resources would help you in this work?
Topic 3: Challenges and barriers: Teaching and learning (T&L)	- Is there anything you find difficult about supporting T&L in your work setting? *What is that? Why is it hard?* - Have you raised this challenge with your manager/colleagues? *Why is this? What response did you get? What do you think of this?* - Have you received any support with this? *Why is that? Was it effective?* - Have you encountered any particular obstacles or barriers in your role of supporting T&L? *Can you give me an example?* - Have you disclosed this obstacle to your manager/colleagues? - How do you overcome this barrier? *Is this effective? Why do you think this?*
Topic 4: Benefits and strengths: Teaching and learning (T&L)	- Have you found there to be any benefits of being autistic when working in T&L - Has being autistic enhanced your T&L? If so, how and can you give examples? - Have you disclosed your autism diagnosis/identity to your colleagues and has this had any positive impacts on your work? if so, can you explain how - Have you encountered any strengths or specific skill sets related to being autistic that you can apply to your role of supporting T&L? Please explain further.
Topic 5: Supports and recommendations	- Is there any support available to you at work? *Are they aware you are autistic? Can you give me an example of available support?* - Do you avail of any of the support offered by the college? *Can you give me an example of the support you receive?* - Do you think that if you had an issue relating to your work/setting, you would have someone to speak to? *Who/where?* - In your opinion, is there anything else your workplace could do to support you in your work? *Can you explain?*

## References

[bibr1-13623613261440799] Baird A. (2020). Teaching while autistic: Constructions of disability, performativity, and identity. Ought: The Journal of Autistic Culture, 2(1), 36–45.

[bibr2-13623613261440799] Botha M. Frost D. M. (2020). Extending the minority stress model to understand mental health problems experienced by the autistic population. Society and Mental Health, 10(1), 20–34.

[bibr3-13623613261440799] Braun V. Clarke V. (2021). Thematic analysis: A practical guide. SAGE Publications.

[bibr4-13623613261440799] Brown N. Leigh J. (2018). Ableism in academia: Where are the disabled and ill academics? Disability & Society, 33(6), 985–989. 10.1080/09687599.2018.1455627

[bibr5-13623613261440799] Cherewick M. Matergia M. (2024). Neurodiversity in practice: A conceptual model of autistic strengths and potential mechanisms of change to support positive mental health and wellbeing in autistic children and adolescents. Advances in Neurodevelopmental Disorders, 8(3), 408–422.

[bibr6-13623613261440799] Doherty M. McCowan S. Shaw S. C. (2023). Autistic SPACE: A novel framework for meeting the needs of autistic people in healthcare settings. British Journal of Hospital Medicine, 84(4), 1–9. 10.12968/hmed.2023.0006 37127416

[bibr7-13623613261440799] Fletcher-Watson S. Adams J. Brook K. Charman T. Crane L. Cusack J. Pellicano E. (2019). Making the future together: Shaping Autism research through meaningful participation. Autism, 23(4), 943–953. 30095277 10.1177/1362361318786721 PMC6512245

[bibr8-13623613261440799] Fletcher-Watson S. Happé F. (2019). Autism: A new introduction to psychological theory and current debate. Routledge.

[bibr9-13623613261440799] Fricker M. (2007). Epistemic injustice: Power and the ethics of knowing. Oxford University Press. 10.1093/acprof:oso/9780198237907.001.0001

[bibr10-13623613261440799] Gormley L. Feeney A. McNally S. (2023). A scoping review of research trends and future directions for research on the experiences of autistic students and staff in post-secondary education. European Journal of Higher Education, 14(4), 554–573. 10.1080/21568235.2023.2231186

[bibr11-13623613261440799] Grove R. Roth I. Hoekstra R. A. (2016). The motivation for special interests in individuals with autism and controls: Development and validation of the special interest motivation scale. Autism Research, 9(6), 677–688. 10.1002/aur.1560 26496939

[bibr12-13623613261440799] Hartman D. O’Donnell-Killen T. Doyle J. K. Kavanagh M. Day A. Azevedo J. (2023). The adult autism assessment handbook: A neurodiversity affirmative approach. Jessica Kingsley Publishers.

[bibr13-13623613261440799] Helsen V. Enzlin P. Gijs L. (2022). Mental health in transgender adults: The role of proximal minority stress, community connectedness, and gender nonconformity. Psychology of Sexual Orientation and Gender Diversity, 9(4), 466–477.

[bibr14-13623613261440799] Higgins J. M. Arnold S. R. Weise J. Pellicano E. Trollor J. N. (2021). Defining autistic burnout through experts by lived experience: Grounded Delphi method investigating #AutisticBurnout. Autism, 25(8), 2356–2369. 10.1177/13623613211019858 34088219

[bibr15-13623613261440799] Huang Y. Hwang Y. I. Arnold S. R. Lawson L. P. Richdale A. L. Trollor J. N. (2022). Autistic adults’ experiences of diagnosis disclosure. Journal of Autism and Developmental Disorders, 52, 5301–5307. 34978025 10.1007/s10803-021-05384-z

[bibr16-13623613261440799] Hull L. Levy L. Lai M.-C. Petrides K. V. Baron-Cohen S. Allison C. Smith P. Mandy W. (2021). Is social camouflaging associated with anxiety and depression in autistic adults? Molecular Autism, 12(1), 13. 10.1186/s13229-021-00421-1 33593423 PMC7885456

[bibr17-13623613261440799] Janse van Rensburg M. Liang B. (2023). Improving autistic students’ experiences in higher education: Developing a community framework for individual autistic student and autistic community flourishing. Autism in Adulthood, 7(2), 141–154. 10.1089/aut.2022.0079 PMC12038307 40309019

[bibr18-13623613261440799] Jones S. C. (2023a). Advice for autistic people considering a career in academia. Autism, 27(7), 2187–2192. 10.1177/13623613231161882 36950875 PMC10504807

[bibr19-13623613261440799] Jones S. C. (2023b). Autistics working in academia: What are the barriers and facilitators? Autism, 27(3), 822–831. 10.1177/13623613221118158 35959515

[bibr20-13623613261440799] Kenny N. Doyle A. (2024). A phenomenological exploration of the lived experience of adults experiencing pathological demand avoidance. Neurodiversity, 2, 27546330241277075.

[bibr21-13623613261440799] Kenny N. Doyle A. Horgan F. (2023). Transformative inclusion: Differentiating qualitative research methods to support participation for individuals with complex communication or cognitive profiles. International Journal of Qualitative Methods, 22, 16094069221146992. 10.1177/16094069221146992

[bibr22-13623613261440799] Kenny N. Neilson S. O’Kelly J. Doyle J. K. McDonald J. (2024). Exploring the paradigm of co-produced research within the context of the COVID-19 pandemic. In Rose R. Shevlin M. (Eds.), Including voices (International perspectives on inclusive education) (Vol. 23, pp. 149–161). Emerald Publishing Limited. 10.1108/S1479-363620240000023012

[bibr23-13623613261440799] Kourti M. (2021). A critical realist approach on Autism: Ontological and epistemological implications for knowledge production in autism research. Frontiers in Psychology, 12, 713423. 10.3389/fpsyg.2021.713423 PMC8732992 35002826

[bibr24-13623613261440799] Leedham A. Thompson A. R. Smith R. Freeth M. (2020). “I was exhausted trying to figure it out”: The experiences of females receiving an autism diagnosis in middle to late adulthood. Autism, 24(1), 135–146. 31144507 10.1177/1362361319853442

[bibr25-13623613261440799] Lewis L. F. (2017). A mixed methods study of barriers to formal diagnosis of autism spectrum disorder in adults. Journal of Autism and Developmental Disorders, 47(8), 2410–2424. 10.1007/s10803-017-3168-3 28516422

[bibr26-13623613261440799] McCoy S. Ye K. Carroll E. (2025). Paths, tracks, gaps and cliffs: The post-school transitions of students with special educational needs. National Council for Special Education. https://www.esri.ie/publications/paths-tracks-gaps-and-cliffs-the-post-school-transitions-of-students-with-special

[bibr27-13623613261440799] McGoldrick E. Munroe A. Ferguson R. Byrne C. Doherty M. (2025). Autistic SPACE for Inclusive Education. Neurodiversity, 3, 1–15. 10.1177/27546330251370655

[bibr28-13623613261440799] Meyer I. H. (1995). Minority stress and mental health in gay men. Journal of Health and Social Behavior, 36(1), 38–56. 7738327

[bibr29-13623613261440799] Milton D. E. (2012). On the ontological status of autism: The “double empathy problem”. Disability & Society, 27(6), 883–887.

[bibr30-13623613261440799] Neilson S. O’Kelly J. Doyle J. K. Kenny N. O’Neill C. Butler S. McDonald J. (2025). “. . . Fallen through the cracks . . .”: A co-produced qualitative exploration of autistic student experiences at an Irish Higher Education Institution. Autism in Adulthood, 7(4), 505–516. http://doi.org/10.1177/25739581251362835 40933683 10.1177/25739581251362835 PMC12417852

[bibr31-13623613261440799] Nicolaidis C. Raymaker D. Kapp S. K. Baggs A. Ashkenazy E. McDonald K. Joyce A. (2019). The AASPIRE practice-based guidelines for the inclusion of autistic adults in research as co-researchers and study participants. Autism, 23(8), 2007–2019. 30939892 10.1177/1362361319830523 PMC6776684

[bibr32-13623613261440799] O’Kelly J. Kenny N. Butler S. Scully C. (2024). Future expectations: A qualitative exploration of the perceptions and expectations of autistic adults regarding the IMPACT programme. Dublin City University. https://drive.google.com/file/d/1B8M0TvPkP7_9_5nIfjK01J5dxzOaJVZA/view

[bibr33-13623613261440799] O’Neill C. Kenny N. (2023). “I saw things through a different lens. . .”: An interpretative phenomenological study of the experiences of autistic teachers in the Irish education system. Education Sciences, 13(7), 670. 10.3390/educsci13070670

[bibr34-13623613261440799] Pearson A. Rose K. (2021). A conceptual analysis of autistic masking: Understanding the narrative of stigma and the illusion of choice. Autism in Adulthood, 3(1), 52–60. 36601266 10.1089/aut.2020.0043 PMC8992880

[bibr35-13623613261440799] Pellicano E. Heyworth M. (2023). The foundations of autistic flourishing. Current Psychiatry Reports, 25(9), 419–427. 10.1007/s11920-023-01441-9 37552401 PMC10506917

[bibr36-13623613261440799] Phung J. Penner M. Pirlot C. Welch C. (2021). What I wish you knew: Insights on burnout, inertia, meltdown, and shutdown from autistic youth. Frontiers in Psychology, 12, Article 741421. 10.3389/fpsyg.2021.741421 PMC8595127 34803822

[bibr37-13623613261440799] Prendeville P. (2025, September 13). Implementing organizational change using SPACE to support autistic and neurodivergent adults with intellectual disability [Oral Presentation]. Autism Europe Congress 2025: Quality of Life–Research, Policy and Practice, Royal Dublin Society, Dublin, Ireland.

[bibr38-13623613261440799] Romualdez A. M. Heasman B. Walker Z. Davies J. Remington A. (2021). “People might understand me better”: Diagnostic disclosure experiences of autistic individuals in the workplace. Autism in Adulthood, 3(2), 157–167.

[bibr39-13623613261440799] Scanlon G. Doyle A. (2021). Transition stories: Voices of school leavers with intellectual disabilities. British Journal of Learning Disabilities, 49(4), 456–466. 10.1111/bld.12433

[bibr40-13623613261440799] Shaw K. A. Williams S. Patrick M. E. Valencia-Prado M. Durkin M. S. Howerton E. M. Ladd-Acosta C. M. Pas E. T. Bakian A. V. Bartholomew P. Nieves-Muñoz N. Sidwell K. Alford A. Bilder D. A. DiRienzo M. Fitzgerald R. T. Furnier S. M. Hudson A. E. Pokoski O. M. . . .Maenner M. J. (2025). Prevalence and early identification of autism spectrum disorder among children aged 4 and 8 years—Autism and Developmental Disabilities Monitoring Network, 16 Sites, United States, 2022. MMWR Surveillance Summaries, 74, 1–22. 10.15585/mmwr.ss7402a1 PMC12011386 40232988

[bibr41-13623613261440799] Sinclair J. (1993). Don’t mourn for us. Autism Network International. https://www.autreat.com/dont_mourn.html

[bibr42-13623613261440799] Sparkes I. Riley E. Cook B. Machuel P. (2022). Outcomes for disabled people in the UK: 2021. Office for National Statistics.

[bibr43-13623613261440799] Stark E. Ali D. Ayre A. Schneider N. Parveen S. Marais K. Holmes N. Pender R. (2021). Coproduction with autistic adults: Reflections from the authentistic research collective. Autism in Adulthood, 3(2), 195–203. 10.1089/aut.2020.0050 36601467 PMC8992895

[bibr44-13623613261440799] Thewissen S. Ozguzel C. Ussing T. M. (2021). Disability, work and inclusion in Ireland: Engaging and supporting employers. Organisation for Economic Co-operation and Development.

[bibr45-13623613261440799] Thompson-Hodgetts S. Labonte C. Mazumder R. Phelan S. (2020). Helpful or harmful? A scoping review of perceptions and outcomes of autism diagnostic disclosure to others. Research in Autism Spectrum Disorders, 77, 101598. 10.1016/j.rasd.2020.101598

[bibr46-13623613261440799] Walker N. (2021). Neuroqueer heresies: Notes on the neurodiversity paradigm, autistic empowerment, and postnormal possibilities. Autonomous Press.

[bibr47-13623613261440799] Walker N. Raymaker D. M. (2021). Toward a neuroqueer future: An interview with Nick Walker. Autism in Adulthood, 3(1), 5–10. 10.1089/aut.2020.29014.njw 36601271 PMC8992885

[bibr48-13623613261440799] Wong B. (2023). Exploring the spatial belonging of students in higher education. Studies in Higher Education, 49(3), 546–558. 10.1080/03075079.2023.2243285

[bibr49-13623613261440799] Wood R. Happé F. (2021). What are the views and experiences of autistic teachers? Findings from an online survey in the UK. Disability & Society, 38(1), 47–72.

